# Impact of Guar Gum and Locust Bean Gum Addition on the Pasting, Rheological Properties, and Freeze–Thaw Stability of Rice Starch Gel

**DOI:** 10.3390/foods11162508

**Published:** 2022-08-19

**Authors:** Xuejiao Xu, Shuhui Ye, Xiaobo Zuo, Sheng Fang

**Affiliations:** 1 College of Biology and Environmental Engineering, Zhejiang Shuren University, Hangzhou 310015, China; 2 School of Food Science and Biotechnology, Zhejiang Gongshang University, Hangzhou 310018, China; 3 Hangzhou Tea Research Institute, CHINA COOP/Zhejiang Key Laboratory of Transboundary Applied Technology for Tea Resources, Hangzhou 310016, China

**Keywords:** rice starch, guar gum, locust bean gum, rheological properties, RVA, freeze–thaw stability

## Abstract

Improving the gel texture and stability of rice starch (RS) by natural hydrocolloids is important for the development of gluten-free starch-based products. In this paper, the effects of guar gum and locust bean gum on the pasting, rheological properties, and freeze–thaw stability of rice starch were investigated by using a rapid visco analyzer, rheometer, and texture analyzer. Both gums can modify the pasting properties, revealed by an increment in the peak, trough, and final viscosities, and prevent the short-term retrogradation tendency of RS. Dynamic viscoelasticity measurements also indicated that the starch–gum system exhibits superior viscoelastic properties compared with starch alone, as revealed by its higher storage modulus (*G*′). Compared with the control, the hysteresis loop area of the guar gum-containing system and locust bean gum-containing system was reduced by 37.7% and 24.2%, respectively, indicating that the addition of gums could enhance shear resistance and structure recovery properties. The thermodynamic properties indicated that both gums retard short-term retrogradation as well as long-term retrogradation of the RS gels. Interestingly, the textural properties and freeze–thaw stability of the RS gel were significantly improved by the addition of galactomannans (*p* < 0.05), and guar gum was more effective than locust bean gum, which may be due to the different mannose to galactose ratio. The results provide alternatives for gluten-free recipes with improved texture properties and freeze–thaw stability.

## 1. Introduction

Rice starch (RS) products are common diets in China and Southeast Asian countries due to the consumer-friendly taste, texture, and ease-of-preparation feature [1,2]. RS products are also an important alternative diet for patients with celiac disease [3]. However, RS gels have deficiencies in ductility, elasticity, and extensibility in the processing and texture of products [4,5]. In addition, RS gel is prone to retrograde during storage, especially during freeze–thaw cycles [6]. Hydrocolloids are widely used in the food industry as functional additives [7,8,9]. Generally, hydrocolloids play critical roles in modifying the rheological and pasting properties of starch, with enhancement in production process efficiency and optimization of texture stability and sensory properties [8,9]. Mechanistic studies have revealed that the incorporation of hydrocolloids in native starches could improve their moisture content and water-holding capacity [10].

Galactomannans are linear hydrocolloids originally found in the endosperm of seeds of various legumes. Studies have revealed that most galactomannans form nonionic structures, which facilitate the formation of functional properties of pH resistance and stability in both ion-enriched solutions and heating processes [11]. In addition, galactomannans are also used as functional ingredients during digestion due to their ability to slow down the degradation rate of starch [12]. Of all the legume seed galactomannans, guar gum and locust bean gum are the most commonly used in the food industry.

Guar gum (*Cyamposis tetragonoloba*) and locust bean gum (*Ceretonia siliqua*) are classical galactomannans comprising a β-(1→4) linked mannopyranosyl linear backbone with α-(l→6) linked D-galactopyranosyl branched chains [13]. Guar gum and locust bean gum have similar molecular structures but differ in the ratio of D-mannosyl: D-galactosyl units, which are 2:1 and 4:1, respectively [14]. Guar gum and/or locust bean gum have been reported to modify the properties of yam starch [15], tapioca starch [16], acorn starch [17], and corn starch [18]. It has been found that guar gum generally exhibits pronounced elastic properties, whereas locust bean gum enhances the viscous properties of tapioca starch owing to the different chain extensions and hydrogen bond numbers [19,20]. Significant improvements in the freeze–thaw stability of corn starch with the incorporation of guar gum rather than that of locust bean gum have been reported, which have contributed to the more frequent interaction between guar gum and leached amylose [15]. Thus, the interactions between hydrocolloids and starch could be crucial for starch-based food products.

It has been reported that the different synergistic effects of starch and hydrocolloids could be related to the physicochemical differences of hydrocolloids such as solubility [21,22,23], intrinsic viscosity [24], extended conformation [25,26], antioxidant potential [27,28], hydrogen bonding capacity [29], flexibility [30], and temperature of gel formation [24,31,32]. The diversity of hydrocolloid molecular structures, especially for the differences in the side chain, usually contributes to the variations in physicochemical properties [33]. Therefore, comprehensive analyses of hydrocolloids and related starch would be beneficial for the further application of related products. However, to the best of our knowledge, the comprehensive understanding of guar gum and locust bean gum when added to rice starch remains unknown [34].

This study aimed to investigate the effect of two galactomannans (guar gum and locust bean gum) on the pasting, rheological properties, and freeze–thaw stability of RS and the possible interaction mechanism. The results may provide valuable information for the selection of gluten-free recipes with improved textural properties and freeze–thaw stability.

## 2. Materials and Methods

### 2.1. Materials

Guar gum (CAS No.: 9000-30-0) and locust bean gum (CAS No.: 9000-40-2) were purchased from Aladdin Industrial Corporation. Rice starch (food-grade) was supplied from Jiangxi Jinnong Co., Ltd. (Yichun, China). The main components of RS were analyzed by standard analytical methods [9]. The total starch content of RS was 90.5 ± 1.35%, the amylose content was 24.67 ± 0.27%, the fat content was 0.05 ± 0.01%, the protein content was 0.90 ± 0.06%, and the ash and moisture content were 0.22 ± 0.02% and 7.7 ± 0.03%, respectively.

### 2.2. Pasting Properties

The pasting properties of RS gels were measured using a Rapid Visco Analyzer (RVA Tec Master, Perten instruments, Hägersten, Sweden) following previous methods [35]. RS alone slurries were obtained by dispersing 3 g RS powder in 25 g distilled water. In the case of RS/gum mixtures, weighed amounts of guar gum or locust bean gum powder were dispersed in distilled water firstly to prepare the hydrocolloid solutions (0.1 wt.%), with an hour of magnetic stirring at 80 °C. Then, 3 g RS powder was dispersed in the prepared solution (ca. 25 g) with mild stirring to obtain the mixture. The prepared slurries weighing 28 g were then transferred to aluminum RVA canisters to investigate the pasting properties. The starch slurry was at first held at 50 °C for 1 min, then heated to 95 °C within 3 min 45 s and maintained at 95 °C for 2 min 30 s. It was subsequently cooled to 50 °C within 3 min 45 s and maintained at 50 °C for 1 min 30 s. For the first 10 s before measurement, the speed of the plastic paddle was set as 960 rad/s for complete dispersion and then kept constant at 160 rad/s during measurement. RVA parameters such as peak viscosity (PV), trough viscosity (TV), final viscosity (FV), breakdown value (BV), pasting temperature (PT), and setback value (SBV) were obtained from the pasting curves. A pan containing the same mass of distilled water was used as a reference. RVA parameters were presented as the mean ± SD of triplicate experiments.

### 2.3. Rheological Properties

The rheological behaviors of RS gels were determined by using an AR-G2 rheometer (TA Instruments, New Castle, DE, USA) with 40 mm parallel plates according to previous methods [33]. The gelatinized slurries obtained from RVA analysis were then immediately transferred to the platform of the rheometer. Before testing, all samples were equilibrated at 25 °C for 120 s and then examined by both dynamic viscoelastic and steady flow measurements.

Dynamic viscoelastic tests were conducted in a frequency range from 0.1 to 10 Hz with a constant strain of 1%. The dynamic rheological data storage modulus (*G*′) and loss modulus (*G*″) vs. angular frequency (ω) were obtained and analyzed by using the following equations [36]:

*G*′ = *K*′(ω)*^n^*^′^ (1)

*G*″ = *K*″(ω)*^n^*^″^ (2)

where *K*′ is constant, ω is the angular frequency, and *n*′ is the frequency exponents.

Next, the steady flow tests were performed, and the shear rate as a function of shear stress was obtained by using the Data software. The shear rate ramps from 0.01 to 300 s^−1^ (upward flow curve) and then decreases from 300 s^−1^ to 0.01 s^−1^ after 1 min of equilibration (downward flow curve). Experiment results from the ascending and descending segments of the shear cycle were then fitted using the power-law model to characterize the flow properties [36]:

(3) $$\sigma =K\times \gamma^{n}$$

where *σ* is the shear stress (Pa), *K* is the consistency coefficient (Pa·s^n^), *γ* is the shear rate (s^−1^), and *n* is the flow behavior index (dimensionless).

### 2.4. Thermodynamic Properties

Thermodynamic properties of the RS gels in the presence or absence of gums were determined by using a C80 differential scanning calorimeter (Setaram, Lyon, France) following previous methods with some modification [37]. Samples were prepared by dispersing 5 g RS powder in 15 g distilled water or hydrocolloid solutions (0.15 wt.%) and stirred for 2 h. Thereafter, the well-stirred suspensions (ca. 4 g) were transferred to an aluminum crucible and tested. The heating temperature ramped from 30 to 100 °C with an acceleration of 0.5 °C/min, and then the ramp was reversed to 30 °C at a rate of −2 °C/min. The instrument was calibrated by using indium and an empty pan as reference. After gelatinization, samples were cooled down and stored at 4 °C for 0, 3, 5, and 12 days, then heated again to investigate the effect of guar gum and locust bean gum on the retrogradation of RS gels. The retrogradation degree was calculated by the ratio of retrogradation enthalpy in the second run heating (Δ*H*_2_) to the gelatinization enthalpy in the first run test (Δ*H*_1_) [38].

### 2.5. Texture Properties

The textural properties of the RS in the presence or absence of gums were determined by using a TA-XTplus Texture Analyzer (Stable Micro System Ltd., Godalming, UK). The gel samples were prepared as described in Section 2.1 and stored at 4 °C for 0, 3, and 5 d. After being conditioned at room temperature, the samples were analyzed by using texture profile analysis (TPA). The TPA tests were performed on samples 25 mm in diameter and 20 mm in height. The sample was placed on the text platform and squeezed twice to 15 mm with a 25.0 mm diameter cylinder probe P/25. The speed of the probe was 3 mm/s, and the trigger force was 5 g. The interval between two compressions was 3 s, and the data acquisition rate was 200 pps.

### 2.6. Freeze–Thaw Stability

The freeze–thaw stability of RS in the presence or absence of gums was determined following the method of Zhai et al. [39] with some modifications. Samples were prepared as described in Section 2.2. The slurry was cooled to 30 °C and then transferred to a preweighed centrifuge tube (10 mL) to record its total weight. The samples were then frozen at −21 °C for 24 h, thawed at 30 °C for 2 h, and centrifuged at 8000 rpm for 20 min. The supernatant was discarded and then weighed. These steps were repeated five times to determine the freeze–thaw stability of samples. The syneresis rate was calculated from the following Equation (4):

(4) $$\mathrm{Syneresis}\;(\%)=\frac{\left(M_{2}-M_{3}\right)\times 100}{M_{2}-M_{1}}$$

where *M*_1_ (g) is the weight of the centrifugal tube, *M*_2_ (g) is the weight of starch paste and centrifuge tube, and *M*_3_ (g) is the weight of starch paste and centrifuge tube after pouring out the supernatant.

### 2.7. Statistical Analysis

The experimental data were analyzed by variance analysis for a completely random design using the SPSS package (19.0, SPSS Inc., Armonk, NY, USA). Duncan’s multiple range tests were conducted to analyze the difference between means with a statistical significance of *p* < 0.05.

## 3. Results and Discussion

### 3.1. Pasting Properties

**Figure 1:**
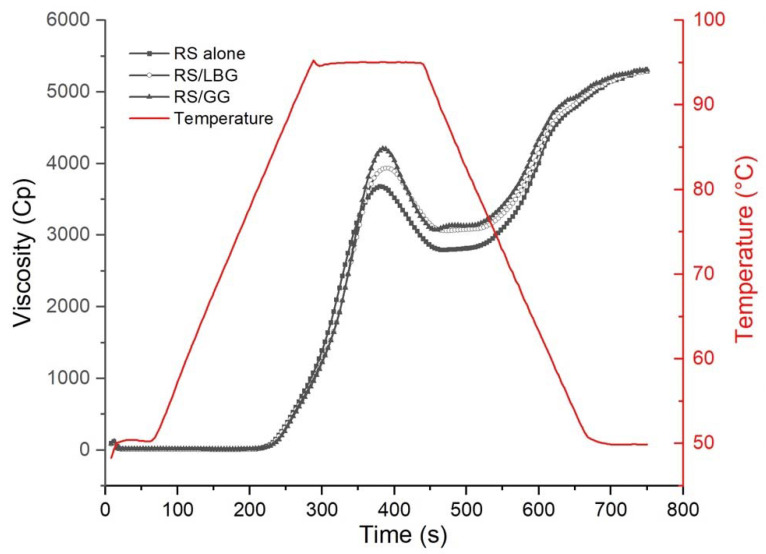
RVA pasting curves of RS alone, RS containing locust bean gum (RS/LBG), and RS containing guar gum (RS/GG).

The pasting curves of RS in the presence or absence of guar gum or locust bean gum are shown in Figure 1. According to the results of pasting behaviors (Table 1), a significant increment was determined in peak and trough viscosity in the presence of galactomannans (*p* < 0.05). This observation was interpreted by the thickening properties of galactomannans. Guar gum and locust bean gum would enhance the shearing forces exerted on the starch granules [40] and defer the hydrolysis rate of starch [41], which is related to the higher viscosity. Moreover, the effective starch concentration was increased by the immobilization of the water molecules [11], enhancing a strong entanglement with amylose and hydrocolloids [24]. Particularly, the viscosity increment of the starch system with guar gum was more pronounced than that with locust bean gum. The primary and secondary OH (hydroxyl groups) located at the exterior branch of guar gum preferred to form more hydrogen bonds, which exhibited the highest PV and BV in the starch-related system.

The breakdown viscosity (BV) of the RS mixture ranged between 878.33 and 1137.33 cP. Higher BV was observed in the guar gum-related system, which indicated its relatively lower thermostability and compatibility compared with the locust bean gum. Similar observations have been obtained in that the BV of wheat flour pastes was increased along with an increment in guar gum levels [41].

SBV could be used as an indicator to measure the syneresis level of starch during the cooling process [42]. Starch systems containing both guar gum and locust bean gum exhibited a lower tendency to retrograde, as evidenced by the lower SBV value. The extension of the network structure in the paste system was inhibited due to the interaction between the colloidal molecules and the leached amylose [43]. Meanwhile, the addition of strong hydrophilic colloids could reduce the free water content, thereby hindering the rearrangement of starch.

### 3.2. Dynamic Viscoelastic Properties

**Figure 2:**
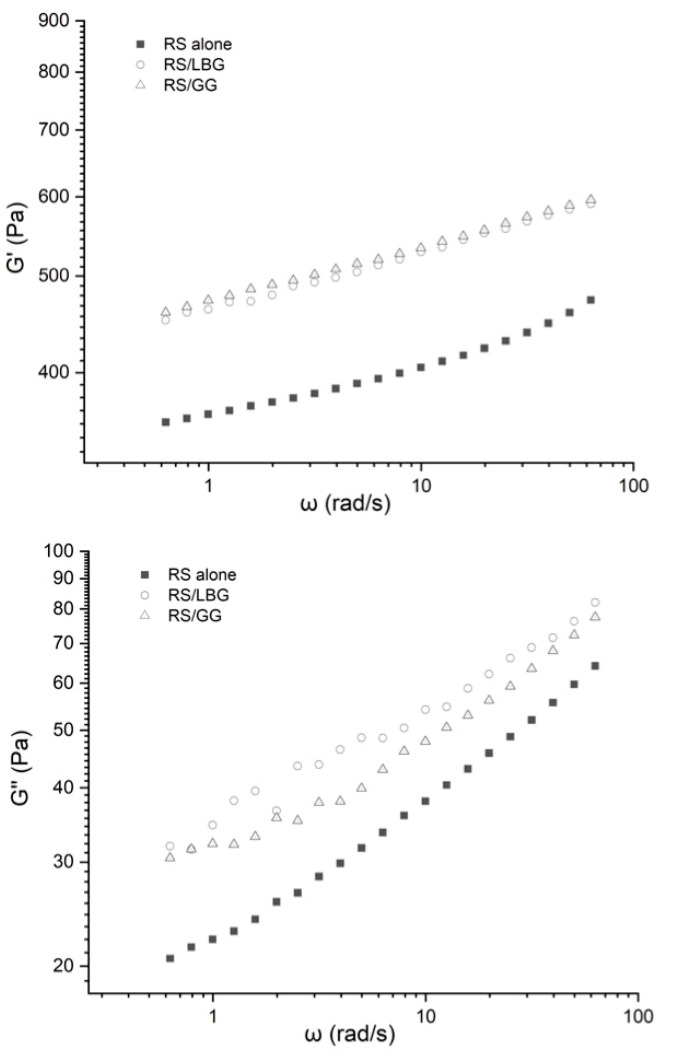
Storage modulus and loss modulus of RS alone, RS containing locust bean gum (RS/LBG), and RS containing guar gum (RS/GG).

The storage modulus (*G*′) and loss modulus (*G*″) of RS gels in the presence or absence of gums are shown in Figure 2. It shows that the values of *G*′ and *G*″ increased with an increasing angular frequency for all samples. Similar results have also been reported with other starch-related systems [44]. In addition, a significant increment in *G*′ and *G*″ was determined with the addition of galactomannans. For example, the *G*′ values of the guar gum-containing system increased from 356.9 to 459.9 Pa with respect to the control. The viscoelastic properties of starch-related systems were enhanced owing to the thickening properties of galactomannans [45].

Interestingly, it seemed different hydrocolloids modified the elastic and viscous properties of starch systems variously. For the guar gum-containing system, the increasing rate of the *G*′ value was much greater than that of the *G*″ value, indicating remarkable elastic properties that could be considered an enhancement of the weak gel network due to more OH groups on the galactose chain. It has been reported that more galactose substitutions of guar gum would hinder intramolecular hydrogen bond formation, thereby exhibiting a more extended conformation than locust bean gum [20]. It is known that noncovalent intermolecular interactions such as hydrogen bonding are the most common and important interaction types between biopolymers [46,47]. It is predicted that the extended chain form promoted the interaction between amylose and guar gum via a noncovalent bond, thereby enhancing the elasticity and pseudo-plasticity of the system [39,48]. On the contrary, the locust bean gum-containing system exhibited pronounced viscous properties, as evidenced by the higher tan(*δ*) and *G*″ values (Table 2). A similar trend has also been reported with a corn starch–galactomannan system [17].

For all RS-related systems, ln(*G*′, *G*″) as a function of ln ω were conducted using linear regression and are summarized in Table 3. It was observed that each starch-related system exhibited weak gel-like behavior with positive slopes (*n*′ and *n*″) [48]. The value of *K*′ and *K*″ increased significantly after the addition of galactomannans, indicating enhanced viscoelasticity of the continuous phase caused by the thickening properties of gums. Such observation is in agreement with maize starch–guar gum mixtures [45] and rice starch–xanthan gum mixtures [34]. The presence of galactomannans facilitated the associations of ordered chain segments, thereby enhancing the weak three-dimensional network of RS–gum mixtures [49].

### 3.3. Steady Shear Properties

**Figure 3:**
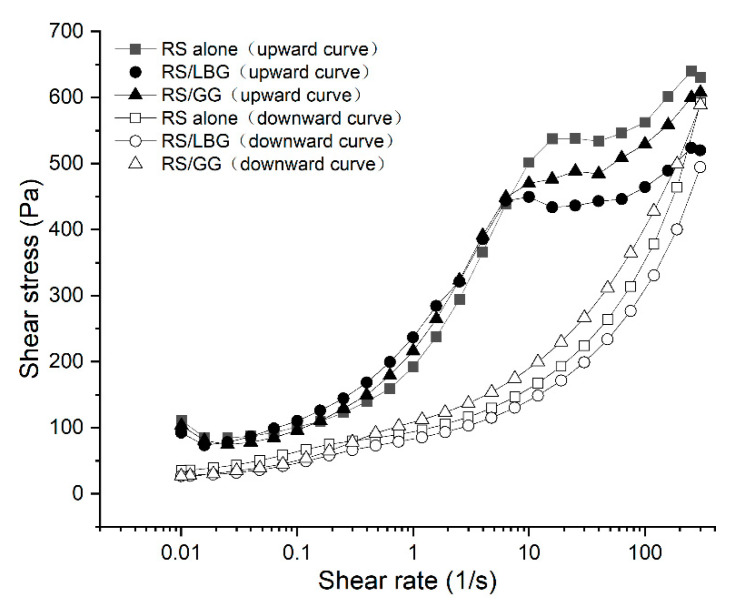
Steady flow curves of RS alone, RS containing locust bean gum (RS/LBG), and RS containing guar gum (RS/GG).

The effects of gums on the steady shear properties of RS gels are shown in Figure 3. Thixotropic behaviors of all samples were observed within the range of shear rates (0.01–300 s^−1^). The first peak that appeared in the upward flow curves was related to the stress required to break the gel structure and caused the solution to recover. Similar results have been reported in the RS–glucans system [38]. The collected data showed a good fit to the power-law model with *R*^2^ between 0.976 and 0.997. For all samples, the consistency coefficient (*K*), the yield stresses (***σ***), flow behavior indices (*n*), and the apparent viscosity at 300 s^−1^(*η*_a, 300_), as well as hysteresis loop areas between the upward and downward curves are summarized in Table 4. Obvious hysteresis loop areas were observed in all starch-related systems, which could be explained by the structural breakdown of the shear field to change the original structure or build up a new one, which then maintained the shear-thinning properties in subsequent shear sweeps [50]. The decomposition of the original structures was observed during gel shearing, as evidenced by *n* < 1. After shearing, the gel structure could only be partially recovered, which is related to the lower *η*_a, 300_ values of the downward curves compared with the upward ones [51]. For the downward curve, it showed that the addition of both gums markedly increased the *K* values, which reflects that the gums mainly enhanced viscoelastic properties, especially for guar gum, owing to the thickening effect.

Compared with the control, the gum-containing systems exhibited more pseudoplastic properties, as evidenced by the higher *K* values (Table 4). Specifically, this effect was more pronounced for the RS–guar gum systems than the RS–locust bean gum systems. This observation is in agreement with the creep recovery results, showing that the guar gum-containing system exhibited higher shear resistance (please see Supplementary Materials Figure S1).

The hysteresis loop area is an indicator to value the structural breakdown during shearing [52]. Compared with the control, the hysteresis loop area of RS gels containing guar gum and locust bean gum were reduced by 37.7% and 24.2%, respectively, indicating remarkable shear resistance, which has been attributed to the enhancement of the three-dimensional structure of the starch [53]. A similar trend was observed in the dynamic viscoelastic results (Figure 2), which, in turn, reflected the retrogradation extent of RS gels (Table 6).

### 3.4. Thermodynamic Properties

Various information such as the starch–hydrocolloid interaction, the effects of water, and related properties could be provided by using DSC analysis. The thermodynamic properties of RS gels in the presence or absence of gums and their corresponding retrograded gels during refrigerated storage (4 °C for 3, 5, and 12 days) are listed in Table 5.

Starch–galactomannan interactions affected the retrogradation of samples. The gelatinization enthalpies (Δ*H*_1_) of the starch system, when added with gums, were slightly increased at first, which may be related to the higher viscosity. In the third and last run, the retrogradation enthalpies (Δ*H*_2_) and retrogradation ratios (Δ*H*_2_/Δ*H*_1_) of retrograded RS gels decreased significantly in the presence of both guar gum and locust bean gum, indicating that gums slowed the retrogradation rate of RS gels during refrigerated storage. Starch retrogradation is generally considered a liquid state event, which requires orientational mobility of the polymer chains in the amylopectin molecule [54]. The presence of guar gum and locust bean gum decreased the orientational mobility of the starch gels and, thus, decreased the retrogradation rate. Moreover, the addition of hydrocolloids could hinder the formation of spongy structures in the starch system, where the gum combined with the starch separated from the starch granules.

The reduction in free water content would inhibit the rearrangement of starch chains, thereby effectively suppressing retrogradation [43]. Interestingly, it seems guar gum suppressed, more than locust bean gum, the short-term retrogradation of RS samples stored for the first 5 days, which was attributed to lower water availability due to the stronger hydration capacity of guar gum [55]. Instead, locust bean gum exhibited the potential to suppress long-term retrogradation, which may be related to its viscous properties, as mentioned above.

The melting enthalpy of recrystallized starch was lower than that of gelatinization, which is consistent with the easier melting properties of recrystallized starch than that of native starch granules [56]. It also found that guar gum and locust bean gum increased or decreased the melting temperatures of recrystallized starch differently during storage time (please see Supplementary Materials Table S1). Similar research [57] has been reported in gum–tapioca starch systems. The results indicated that the addition of guar gum and locust bean gum could modify the thermal properties of RS dependent on the gum type and storage time.

### 3.5. Determination of Texture Properties

The texture properties of RS gels in the presence or absence of gums during refrigerated storage are presented in Table 6. The hardness of starch gel is always an indicator of the degree of starch retrogradation [58]. The higher hardness values of the starch gel during the initial 3 days indicated its rapid retrogradation. This observation is similar to the retrogradation of amaranth starch in that retrogradation is accelerated at refrigerated temperatures [59]. As a result, a more compact and ordered structure is formed by the amylose and amylopectin in the gelatinized starch, thereby increasing the gel hardness of the RS [60].

Both guar gum and locust bean gum exhibited excellent potential in the texture modification of RS products during cold storage time. The numerical reduction in hardness was observed with the addition of gums after 3 days as compared with the control. This observation could be interpreted as the possible interaction between gums and the RS granules. The presence of galactomannans tends to combine with RS molecules, thereby inhibiting the leaching of amylose and hindering the retrogradation of starch-related systems [61]. The arrangement of leached amylose is also retarded by the combination, which further suppresses the recrystallization of starch molecules. Generally, the addition of hydrocolloids would improve water retention and inhibit the rearrangement of amylose of starch, which helps to maintain better texture characteristics during refrigerated storage [62].

To specify, the guar gum-containing system exhibited better textural properties during cold storage time, as shown in Table 6. It has also been reported that hydrogen bonding plays a critical role in the gelatinization and retrogradation of starch [11]. A large amount of hydroxyl groups in guar gum, rather than locust bean gum, tends to transform the nucleus of starch recrystallization from amylose to an amylose–gum mixture. As a result, the arrangement of starch molecules is retarded, leading to a lower retrogradation rate of starch [63]. In general, the hardness of RS alone gel would be increased during cold storage owing to starch retrogradation. Meanwhile, the addition of the gums to RS gels helped to slow down the changes in textural characteristics during refrigerated storage in the order of guar gum > locust bean gum.

### 3.6. Freeze–Thaw Stability

**Figure 4:**
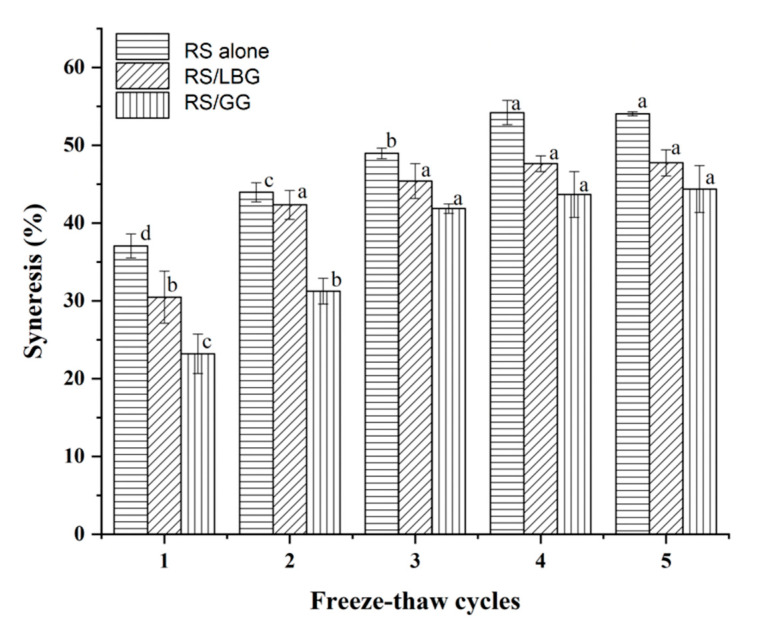
Freeze–thaw stability of RS alone, RS containing locust bean gum (RS/LBG), and RS containing guar gum (RS/GG). Different letters indicate significant differences among group (*p* < 0.05 by Duncan’s multiple range test).

The syneresis value of freeze–thaw RS is often determined as an indicator to evaluate its ability to maintain desirable physical properties during the freezing and thawing process [64,65]. The freeze–thaw stability of RS in the presence or absence of locust bean gum or guar gum is shown in Figure 4. For the RS gels alone, a high syneresis value (37.2%) was observed after the first freeze–thaw cycle. With an increase in freeze–thaw cycles, the syneresis values of gels increased consequently. During repeated freeze–thaw cycles, the starch molecules will recombine, coagulate, and even form a spongy structure, and water will precipitate from the starch body, resulting in dehydration and condensation [24,66].

The evidence is demonstrated by the lower syneresis value of the RS/gums gels than of the control, showing better freeze–thaw stability in the gels containing gums during freeze–thaw cycles. It could be interpreted by the thickening properties of guar gum and locust bean gum, which would reduce the ice crystal size [43]. The presence of hydrocolloids in starch gel could also bind to water molecules, which reduces the syneresis degree during the freeze–thaw cycles [67].

Compared with locust bean gum, guar gum exhibit better stability during all the freeze–thaw cycles. This is consistent with previous research in which guar gum showed pronounced freeze–thaw stability and a more remarkable synergistic combination with sweat potato starch [43] and corn starch [15]. Although locust bean gum showed similar gelatinization viscosity (Table 1), it was not as effective as guar gum. This is because of their different physical structures [68], especially the different ratios of galactose branching to the mannose backbone [69]. For the locust bean gum-containing system, more bulky phase water would be generated during the repeated FT cycles owing to the ease in the chain association [43].

## 4. Conclusions

In the present study, the effects of guar gum and locust bean gum on the gelatinization properties, rheological properties, and freeze–thaw stability of RS gels were investigated. RVA results showed that both guar gum and locust bean gum had inhibitory effects on the retrogradation of RS, with a lower SBV value and higher PV. Higher viscoelastic behavior and less thixotropic behavior were reviewed in the presence of galactomannans in the rheological measurements, which related to the enhanced weak gel structure and higher shear resistance. The thermal properties of RS gels could be modified with the addition of galactomannans, which were dependent on the gum type and storage time. Moreover, the textural properties and freeze–thaw stability of the RS gel were significantly improved by the addition of galactomannans. Particularly, the guar gum-containing system exhibits a more significant effect rather than that of locust bean gum, which could be attributed to the different mannose to galactose ratios. From these findings, guar gum could be a better alternative for gluten-free products, as it confers a higher retrogradation inhibition effect, freeze–thaw stability, and fewer texture changes in gels. Future attempts with more detailed parameters optimization are strongly required to unveil the possible interaction mechanism between hydrocolloids and starch and, finally, pinpoint their effects on human digestibility and the sensory properties of food products.

## Figures and Tables

**Table 1:** Pasting parameters of RS alone, RS containing locust bean gum (RS/LBG), and RS containing guar gum (RS/GG) *.

Sample	PV (cP)	TV (cP)	BV (cP)	FV (cP)	SBV (cP)	PT (°C)
RS alone	3679.67 ± 17.47 ^c^	2790.67 ± 8.33 ^b^	889.00 ± 19.29 ^b^	5287.33 ± 25.89 ^a^	2496.67 ± 26.50 ^a^	82.43 ± 0.42 ^b^
RS/LBG	3940.33 ± 4.04 ^b^	3062.00 ± 23.90 ^a^	878.33 ± 21.03 ^b^	5291.67 ± 7.23 ^a^	2229.67 ± 24.17 ^b^	82.42 ± 0.49 ^b^
RS/GG	4210.00 ± 36.29 ^a^	3072.67 ± 43.68 ^a^	1137.33 ± 22.81 ^a^	5308.67 ± 10.69 ^a^	2236.00 ± 39.00 ^b^	83.25 ± 0.43 ^a^

* Each value represents the mean ± SD of triplicate experiments. Different superscripts with the same columns are significantly different (*p* < 0.05 by Duncan’s multiple range test).

**Table 2:** Storage (*G*′), loss moduli (*G*″), and loss tangent (tan δ) at 6.28 rad s^−1^ for RS alone, RS containing locust bean gum (RS/LBG), and RS containing guar gum (RS/GG) *.

Sample	*G*′ (Pa)	*G*″ (Pa)	tan(*δ*)
RS alone	394.47 ± 21.27 ^b^	33.65 ± 1.35 ^c^	0.085 ± 0.003 ^b^
RS/LBG	512.77 ± 27.98 ^a^	48.51 ± 0.41^a^	0.095 ± 0.006 ^a^
RS/GG	519.60 ± 30.47 ^a^	42.95 ± 1.63 ^b^	0.083 ± 0.005 ^b^

* Mean ± SD. Different superscripts with the same columns are significantly different (*p* < 0.05 by Duncan’s multiple range test).

**Table 3:** Parameters of RS alone, RS containing locust bean gum (RS/LBG), and RS containing guar gum (RS/GG) at 25 °C as determined from Equations (1) and (2) *.

Sample	Storage Modulus *G*′			Loss Modulus *G*″		
	*k*′(pa·s^n^)	*n*″	*R* ^2^	*k*″ (pa·s^n^)	*n*″	*R* ^2^
RS alone	358.76 ± 20.01 ^b^	0.06 ± 0.00 ^a^	0.970	21.25 ± 0.66 ^c^	0.26 ± 0.01 ^a^	0.996
RS/LBG	461.77 ± 28.63 ^a^	0.06 ± 0.00 ^a^	0.997	34.29 ± 0.42 ^a^	0.20 ± 0.00 ^b^	0.988
RS/GG	470.98 ± 33.50 ^a^	0.06 ± 0.01 ^a^	0.998	29.69 ± 1.40 ^b^	0.22 ± 0.01 ^c^	0.974

* Mean ± SD. Different superscripts with the same columns are significantly different (*p* < 0.05 by Duncan’s multiple range test).

**Table 4:** The steady flow fitting parameters of RS alone, RS containing locust bean gum (RS/LBG), and RS containing guar gum (RS/GG) *.

Sample	Hla (Pa·s)	Upward Curve				Downward Curve			
		*K* (Pa·s^n^)	*n*	*η*_a,300_ (Pa·s)	*R* ^2^	*K* (Pa·s^n^)	*n*	*η*_a,300_ (Pa·s)	*R* ^2^
RS alone	53,245	236.57 ± 2.41 ^a^	0.187 ± 0.004 ^ab^	2.15 ± 0.05 ^a^	0.909	81.84 ± 1.6 ^c^	0.334 ± 0.002 ^a^	2.02 ± 0.03 ^a^	0.976
RS/LBG	40,385	236.51 ± 32.32 ^a^	0.152 ± 0.056 ^b^	2.23 ± 0.03 ^a^	0.915	86.64 ± 6.6 ^b^	0.328 ± 0.004 ^b^	2.15 ± 0.02 ^a^	0.987
RS/GG	33,150	237.62 ± 16.60 ^a^	0.180 ± 0.033 ^ab^	2.02 ± 0.56 ^a^	0.951	101.9 ± 12.6 ^a^	0.312 ± 0.002 ^c^	1.96 ± 0.54 ^a^	0.997

* Hla: hysteresis loop area (Pa·s); power-law parameters: *K*, consistency coefficient; n, flow behavior index; *η***_a,300,_** the apparent viscosity at 300 s^−1^. Different superscript letters with the same columns are significantly different (*p* < 0.05 by Duncan’s multiple range test).

**Table 5:** The retrogradation thermodynamic parameters of RS alone, RS containing locust bean gum (RS/LBG), and RS containing guar gum (RS/GG) *.

Samples	First Run	Second Run (3 d at 4 °C)		Third Run (5 d at 4 °C)		Fourth Run (12 d at 4 °C)	
	Δ*H*_1_	Δ*H*_2_	Δ*H*_2_/Δ*H*_1_	Δ*H*_3_	Δ*H*_3_/Δ*H*_1_	Δ*H*_4_	Δ*H*_4_/Δ*H*_1_
RS alone	2.66	0.30	0.11	1.48	0.56	2.32	0.87
RS/LBG	2.76	0.40	0.14	1.38	0.50	1.43	0.52
RS/GG	2.74	0.33	0.12	1.11	0.41	1.47	0.54

* Δ*H*_1_, gelatinization enthalpy; Δ*H*_2_, retrogradation enthalpy; Δ*H*_2_/Δ*H*_1_, retrogradation ratio.

**Table 6:** TPA texture parameters of RS alone, RS containing locust bean gum (RS/LBG), and RS containing guar gum (RS/GG) during cold storage *.

Sample	Days	Hardness	Springiness	Adhesiveness	Cohesiveness	Gumminess	Chewiness
RS alone	3	1134.16 ± 58.91 ^a^	0.37 ± 0.02 ^a^	77.64 ± 4.16 ^b^	0.24 ± 0.02 ^a^	269.40 ± 38.87 ^a^	99.06 ± 20.13 ^a^
	5	907.52 ± 6.93 ^a^	0.26 ± 0.01 ^a^	17.63 ± 3.21 ^a^	0.1 ± 0.01 ^a^	94.42 ± 5.81 ^a^	24.80 ± 2.97 ^a^
RS/LBG	3	931.82 ± 14.62 ^b^	0.36 ± 0.05 ^a^	123.20 ± 14.03 ^a^	0.25 ± 0.02 ^a^	233.56 ± 24.78 ^a^	84.93 ± 20.51^a^
	5	861.36 ± 71.83 ^a^	0.32 ± 0.05 ^a^	11.90 ± 2.06 ^a^	0.10 ± 0.01 ^a^	84.02 ± 1.88 ^a^	26.73 ± 3.90 ^a^
RS/GG	3	861.02 ± 66.74 ^b^	0.32 ± 0.01 ^a^	54.32 ± 5.98 ^c^	0.24 ± 0.04 ^a^	202.81 ± 19.53 ^b^	64.49 ± 4.68 ^a^
	5	762.69 ± 0.54 ^b^	0.28 ± 0.04 ^a^	18.66 ± 4.97 ^a^	0.10± 0.02 ^a^	79.81 ± 13.04 ^a^	21.81 ± 0.23 ^a^

* Values represent the mean ± SD of triplicate tests. Columns with different superscripts are significantly different during different samples (*p* < 0.05 by Duncan’s multiple range test).

## Data Availability

The data presented in this study are available on request from the corresponding author.

## Supplementary Materials

The following supporting information can be downloaded at: https://www.mdpi.com/article/10.3390/foods11162508/s1, Figure S1: The creep-recovery curves of RS alone, RS containing locust bean gum (RS/LBG) and RS containing guar gum (RS/GG); Table S1: Gelatinization temperature and enthalpy and retrogradation ratio for RS alone, RS containing locust bean gum (RS/LBG) and RS containing guar gum (RS/GG) measured by the differential scanning calorimeter (DSC).
