# Supplementary material for: Impact of Guar Gum and Locust Bean Gum Addition on the Pasting, Rheological Properties, and Freeze–Thaw Stability of Rice Starch Gel
Source: Foods. 2022 Aug 19;11(16):2508. doi: 10.3390/foods11162508 (PMC9407422; doi:10.3390/foods11162508)
Supplement: Supplementary file 1 [file foods-11-02508-s001.zip › foods-1820235-supplementary.pdf]

SUPPLEMENTARY MATERIALS

Table S1. Gelatinization temperature and enthalpy and retrogradation ratio for RS alone, RS containing locust bean gum (RS/LBG) and RS containing guar gum (RS/GG) measured by the differential scanning calorimeter (DSC)

Samples	Days	T_o(°C)	T_p(°C)	T_c(°C)	ΔH(J/g)	(%)
	3	46.953	54.663	59.432	0.295	11.09
**RS alone**	5	49.660	55.397	64.258	1.481	55.68
	12	52.939	57.172	60.098	2.315	87.03
	3	46.394	52.513	59.998	0.403	14.60
**RS/LBG**	5	46.857	57.778	61.299	1.376	49.86
	12	52.388	57.414	62.614	1.429	51.78
	3	47.731	53.350	63.244	0.329	12.01
**RS/GG**	5	53.103	58.803	63.685	1.105	40.33
	12	48.540	54.170	63.616	1.468	53.58

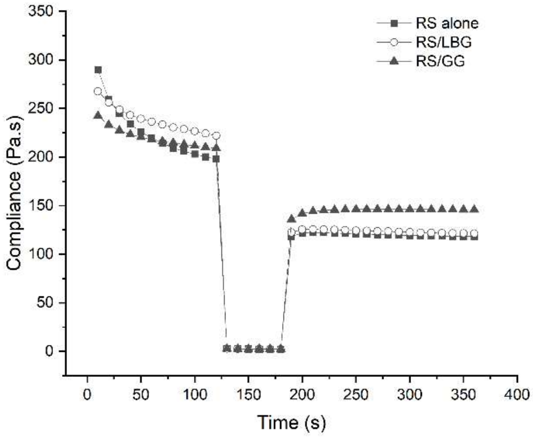

Figure S1. The creep-recovery curves of RS alone, RS containing locust bean gum (RS/LBG) and RS containing guar gum (RS/GG).
